# Osteosarcopenia: A case of geroscience

**DOI:** 10.1002/agm2.12080

**Published:** 2019-09-08

**Authors:** Ben Kirk, Ahmed Al Saedi, Gustavo Duque

**Affiliations:** ^1^ Department of Medicine Western Health Melbourne Medical School University of Melbourne Melbourne Vic. Australia; ^2^ Australian Institute for Musculoskeletal Science (AIMSS) University of Melbourne and Western Health Melbourne Vic. Australia

**Keywords:** bone, cross talk, geroscience, muscle, osteosarcopenia

## Abstract

Many older persons lose their mobility and independence due to multiple diseases occurring simultaneously. Geroscience is aimed at developing innovative approaches to better identify relationships among the biological processes of aging. Osteoporosis and sarcopenia are two of the most prevalent chronic diseases in older people, with both conditions sharing overlapping risk factors and pathogenesis. When occurring together, these diseases form a geriatric syndrome termed “osteosarcopenia,” which increases the risk of frailty, hospitalizations, and death. Findings from basic and clinical sciences aiming to understand osteosarcopenia have provided evidence of this syndrome as a case of geroscience. Genetic, endocrine, and mechanical stimuli, in addition to fat infiltration, sedentarism, and nutritional deficiencies, affect muscle and bone homeostasis to characterize this syndrome. However, research is in its infancy regarding accurate diagnostic markers and effective treatments with dual effects on muscle and bone. To date, resistance exercise remains the most promising strategy to increase muscle and bone mass, while sufficient quantities of protein, vitamin D, calcium, and creatine may preserve these tissues with aging. More recent findings, from rodent models, suggest treating ectopic fat in muscle and bone marrow as a possible avenue to curb osteosarcopenia, although this needs testing in human clinical trials.

## 1 INTRODUCTION

Global aging, attributed to advancements in health care and socioeconomic factors, represents one of the great achievements of the 21st century. However, older age associates with chronic diseases, which could share similar pathophysiology and risk factors; understanding and elucidation of those common mechanisms have enabled the development of geroscience. Musculoskeletal diseases, in particular, represent a significant burden in older persons and a major cost to health systems worldwide. Of those, osteopenia/osteoporosis (characterized by low bone mass) increases with age alongside the number of osteoporotic fractures,^1^ while sarcopenia (low muscle mass and function) confers a high risk of falls and disability in older persons.^2^ Together, these diseases form a geriatric syndrome known as “osteosarcopenia,”^3^ which associates with an increased risk of falls, fractures, and hospitalizations in older persons.^4^, ^5^ Not only does osteosarcopenia induce billions in health‐care expenditure but it also greatly impairs an older person's quality of life.^3^, ^6^

The prevalence of osteosarcopenia in community‐dwelling older adults ranges from 4.7% in Japan,^7^ to 13% in China^8^ and 28% in Germany,^9^ with the highest rates observed in Australia (40%)^3^ and Iran (34%) (N. Fahimfar, unpublished data, June 2019). A study of older Koreans with hip fractures also found that 27.2% were osteosarcopenic.^10^ The varied prevalence is likely due to heterogenous populations or a non‐unified diagnostic criterion for this syndrome, with various screening tools being utilized for low muscle mass and function (sarcopenia), a key component of osteosarcopenia. Irrespective of this, osteosarcopenia confers alarming health‐care costs, which are projected to rise.

Muscle and bone are two highly malleable tissues adapting to environmental stimuli across the life span. Indeed, both tissues develop during adolescence with peak density occurring in the third decade of life, which is largely maintained up until pre‐menopause, and declines thereafter.^11^, ^12^ The interaction and cross talk between muscle and bone have been an increasing area of research in health and disease. These two organs are connected anatomically, as well as through biochemical and biomechanical pathways, and share common risk factors in osteosarcopenia, including epigenetic, endocrine, and mechanical factors.^6^

**Figure 1:**
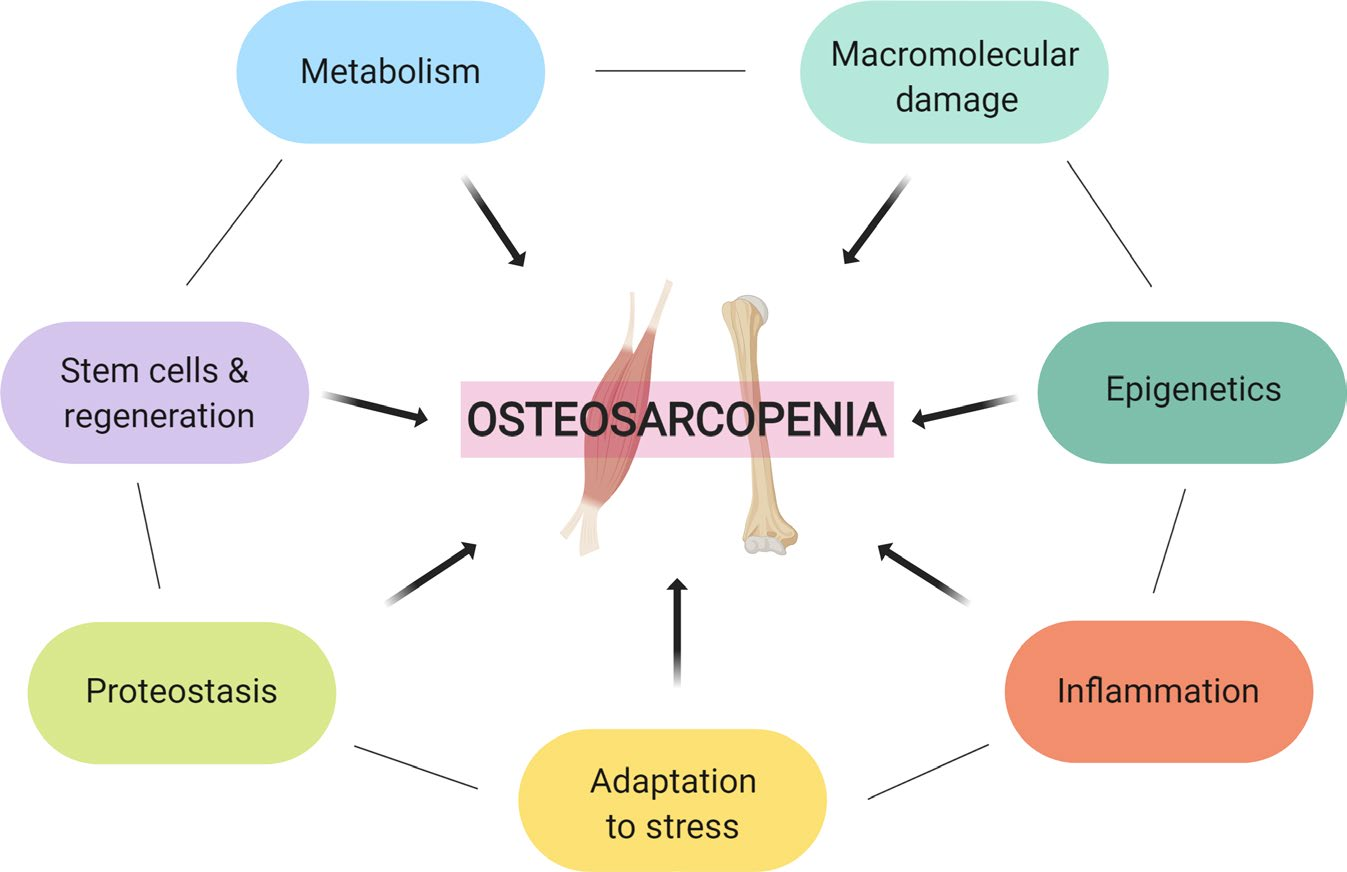
Mechanisms of aging and the development of osteosarcopenia

Given the rapid growth of the aging population, which has led to 13% of the global population aged 65 years and older,^13^ it is critical that those in the field of geroscience appreciate the pathophysiological processes underpinning osteosarcopenia, in order to develop, test, and validate therapeutic strategies targeting both tissues simultaneously. In light of this, we discuss the age‐related alterations to the musculoskeletal system focusing on those “pillars of aging”^14^ that lead to the manifestation of osteosarcopenia (Figure 1).

## 2 PATHOPHYSIOLOGY: PILLARS OF AGING AND OSTEOSARCOPENIA

### 2.1 Muscle and bone mechanics

Muscle attaches to the human skeleton, enabling locomotion, and is a primary source of mechanical stimuli, which generates the strain needed to maintain bone health. This stems from early work indicating that muscle mass accrues more rapidly than bone mass during adolescence, suggesting muscle contraction acts as a stimulus for increases in bone mineral density.^15^ In support, increasing lean (muscle) mass is protective against bone loss and risk of vertebral fractures.^16^ Physical activity also increases muscle and bone volume,^17^, ^18^ while disuse induces atrophy of both tissues.^19^ Moreover, in most but not all studies, muscle and bone mass correlate in aging.^20^ For instance, a 4‐year follow‐up of older Japanese adults found that the prevalence of sarcopenia was high in those with osteoporosis and vice versa.^7^ In the same population, sarcopenic individuals tended to have lower bone density, and those with osteoporosis displayed lower muscle mass and functional capacity.^7^ Interestingly, the presence of osteoporosis was predictive of future risk of sarcopenia (odds ratio, 2.99; 95% confidence interval, 1.46‐6.12), although the opposite relationship was not observed over this 4‐year period.^7^ This finding highlights the need for additional longitudinal trials to investigate if one disease is predictive of the other. This, in turn, will enable further interpretation of the mechanical alterations underlying osteosarcopenia.

As mentioned, in response to environmental stresses, such as loading or unloading, muscle and bone adapt their density and strength.^21^ Due to this biomechanical nature, physical inactivity, along with advancing age, is a primary risk factor for osteoporosis and sarcopenia,^22^ and thus of osteosarcopenia. More recent work in this area has focused on the interactions among genetic, metabolic, and endocrine factors in muscle and bone in the hope of identifying overlapping risk factors for osteosarcopenia.

### 2.2 Genetics and epigenetics

Research into the shared genetic etiology of osteoporosis and sarcopenia shows that approximately 60%‐70% of the risk factors underlying these diseases are heritable,^23^ with both tissues sharing genetic determinants that exert pleiotropic effects. This is due to osteogenic and myogenic cells differentiating from the same mesenchymal precursor. Indeed, young monozygotic twins display a 30%‐45% genetic correlation between lean (muscle) and bone mass at both distal and proximal sites.^24^ Moreover, a recent genetic variant analysis of over 10 000 pediatrics individuals found pleiotropic effects of the sterol regulatory element binding transcription factor 1 (SREBF1), which regulates fat metabolism, is expressed in osteoblasts and myoblasts, and associates with lean mass and bone mineral density.^25^ Several other genetic polymorphisms have been linked to osteosarcopenia, including the genes *GLYAT*, methyltransferase‐like 21C (*METTL21C*), myostatin, α‐actinin 3, proliferator‐activated receptor gamma coactivator 1‐alpha (*PGC‐1*α), and myocyte enhancer factor 2C (*MEF‐2C*).^6^

Thanks to the study of model organisms, epigenetic alterations (including the loss of heterochromatin and core histone proteins), genome instability, DNA methylation, and altered RNA expression have been attributed to cellular senescence.^26^ Epigenetic factors, representing a link between individual genetic aspects and environmental influences, are involved in muscle and bone biology.^27^ Bone and muscle metabolism are under the control of epigenetic mechanisms involving histone deacetylases and microRNAs.^28^, ^29^ Some microRNAs play key roles in the regulation of Wnt signaling in mesenchymal stem cell (MSC) differentiation into myocytes, osteoblasts, and adipocytes.^30^ Most of the current evidence looking at the role of epigenetic mechanisms in muscle and bone development and maintenance has been generated by investigating those tissues separately. Whether epigenetic mechanisms are involved in the pathogenesis of osteosarcopenia, or whether they could become robust biomarkers for this syndrome, is a subject of intense research.

### 2.3 Metabolism

In muscle, protein metabolism is governed by the net balance between protein synthesis and degradation. In the postprandial state, protein synthesis exceeds degradation, while the opposite occurs during periods of energy restriction.^31^ Similarly, bone turnover is regulated by the delicate equilibrium between bone‐forming (osteoblasts) and bone‐resorbing (osteoclasts) cells.^32^ With advancing age, the metabolism of both tissues deteriorates and in situations of inactivity, disuse, or trauma, proteolysis and matrix loss is further exacerbated.^33^ If this imbalance persists and reaches a threshold whereby there is a synergistic loss of bone density, as well as muscle mass, strength, and function, osteosarcopenia occurs.^3^

In parallel with the aging population, the obesity epidemic has resulted in a greater number of older persons with high fat mass. When osteosarcopenia is diagnosed in the presence of obesity, a hazardous duet termed “osteosarcopenic obesity” occurs, which increases the risk of a range of adverse health outcomes.^34^ Independently of the presence of obesity, localized fat infiltration of muscle and bone marrow is now considered a hallmark of aging,^35^, ^36^ degrading surrounding cells, nerves, and capillaries via the secretion of fatty acids and adipokines, which negatively interfere with the cross talk of these organs and subsequently increases fracture risk.^36^

Other pathological conditions, such as diabetes and hyperthyroidism, exacerbate muscle and bone loss. In diabetic states, impaired anabolic signaling is accentuated. In aged muscle, this presents as type II fiber atrophy, insulin resistance, lipotoxicity, decreased glycogen synthesis, and mitochondrial dysfunction.^37^, ^38^ Diabetes is also a secondary cause of osteoporosis and increases fracture risk in osteopenic patients.^39^ Causes include chronic hyperglycemia, advanced glycation end products (AGEs), and oxidative stress, all of which may decrease bone formation.^39^ Moreover, AGEs are known to suppress differentiation of myogenic genes and induce apoptosis,^40^ and others have shown that osteoglycin inhibition impairs myoblast proliferation,^41^ suggesting a shared mechanism via muscle‐bone interactions. Just recently, administration of exogenous insulin‐like growth factor‐1 (IGF‐1) attenuated the detrimental effects of AGEs in myoblastic cells,^40^ perhaps representing a single muscle‐bone‐unit treatment strategy, which warrants further investigation.

Hyperthyroidism also associates with accelerated bone remodeling, reduced bone density, and increased fracture risk.^42^, ^43^ Hyperthyroid patients also present with muscle weakness and impaired Ca^2+^ cycling,^44^ which may explain the increased fatigability observed in hyperthyroid mice.^45^ We have also shown that elevated parathyroid hormone levels associate with poor physical performance and balance in older persons,^46^ which may increase falls risk and is associated with osteosarcopenia.^47^ Glucocorticoid treatment for autoimmune disorders is also common in older patients, but decreases bone remodeling and muscle protein synthesis rates via the ubiquitin/proteasome pathway.^48^ Other risk factors for osteosarcopenia include malnutrition and hyperlipidemia, as well as abnormal hormonal profiles,^6^ which adversely increase or decrease with age.

Other factors influencing muscle and bone metabolism include deficiencies in protein, calcium, and vitamin D, which affect the quality of these tissues.^49^ Low protein intake is associated with low (lean) muscle and bone mass in both cross‐sectional and longitudinal studies.^50^, ^51^, ^52^ Bone also contains the largest storage site of calcium (~99%) in the form of hydroxyapatite; thus, it is unsurprising that appropriate calcium intake remains a widely promoted strategy to prevent osteoporosis.^53^ However, the role of this nutrient in the preservation of bone mass and reduced fracture risk is controversial, with some trials showing benefits^54^, ^55^ and some not.^56^ Calcium also plays a role in cross‐bridge cycling in muscle, with an impairment in Ca^2+^ kinetics linked to a reduction in excitability in sarcopenia,^57^ although it is unknown if low calcium intake influences neuromuscular function via this mechanism.

The role of vitamin D in bone preservation is well established,^55^ and the link between low vitamin D status and muscle wasting and impaired neuromuscular functioning is emerging.^58^, ^59^ Patients with insufficient vitamin D levels exhibit low muscle and bone mass,^60^ and older adults with osteoporosis show type II fiber atrophy,^61^ whereas supplementing with this nutrient is linked to enhancements of lean body mass, strength, and function in sarcopenic older adults.^62^

In vivo work has focused on the vitamin D receptor (expressed in muscle and bone) as a possible mechanism relating to the atrophy of both tissues, with a recent study documenting a reduction in muscle mass and function upon deletion of the vitamin D receptor in myocytes.^63^ An earlier study, again in rodents, also showed that vitamin D deficiency induces muscle wasting and decreased muscle gene markers,^58^ suggesting a role of vitamin D in myogenesis, which may account for the previous findings. Magnesium is another nutrient that has received less attention in musculoskeletal health, despite its role in regulating enzymes involved in calcium metabolism,^64^ a key feature in bone remodeling as well as functioning of the neuromuscular junction. The largest prospective study conducted to date suggests a protective effect of magnesium on osteoporotic fractures;^65^ however, its role in osteosarcopenia warrants further investigation.

### 2.4 Stem cells regeneration and exhaustion

Mesenchymal stem cells are precursors of muscle and bone regeneration and their intrinsic plasticity alters with senescence. Indeed, satellite cell content is reduced in type II myofibers of aged muscle,^66^ and proliferation rates are slowed in comparison to younger muscle.^67^ Observing age‐related changes in myogenesis using electron microscopy demonstrates a decline in myonuclear numbers in older persons.^68^ Furthermore, aged satellite cells are known to translocate across the basal lamina in which they reside at an attenuated rate to initiate myogenesis, a consequence of the age‐related thickening of the extracellular wall.^68^ As such, satellite stem cells are considered a hallmark of sarcopenia by impairing regeneration of myofibers.

On the other hand, bone formation is dependent on the number and activity of osteoblasts during remodeling. Osteoblasts are derived from osteoprogenitor stem cells, residing in the bone marrow. With aging, the proliferation of bone‐marrow stem cells is impaired whereas improving the differentiation of these cells improves bone‐marrow adipogenesis.^69^ Stromal cells from older, compared to younger, rodents show diminished osteogenesis.^70^ Likewise, the osteoblastic effects of adipose‐derived stem cells from older and younger women show impaired differentiation in the former population.^67^ As described, stem cells senescence and exhaustion is an overlapping feature of both sarcopenia and osteoporosis, thus of osteosarcopenia, with purported mechanisms including telomere shortening, increased reactive oxygen species, and reduced transcriptional control.^71^

### 2.5 Cross talking between muscle and bone

**Figure 2:**
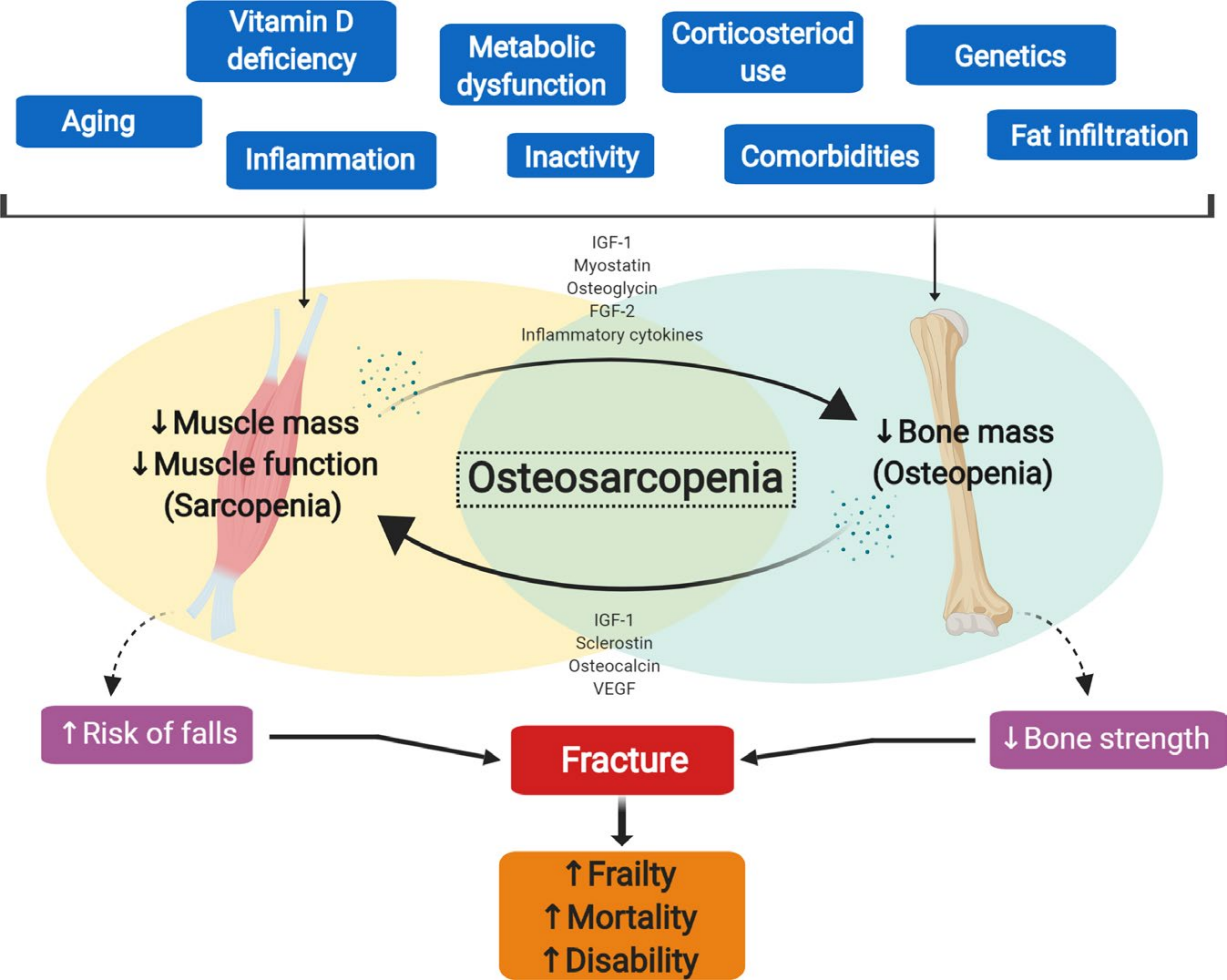
Risk factors, muscle‐bone cross talk, and the pathophysiology of osteosarcopenia. FGF‐2, fibroblast growth factor‐2; IGF‐1, insulin‐like growth factor‐1; VEGF, vascular endothelial growth factor

Historically, the muscle‐bone connection was considered mechanical; however, evidence showing increases in distal, non‐weight‐bearing bones along with muscle mass in response to exercise suggests an endocrine interaction.^72^ This is further evidenced by eloquent work demonstrating an acceleration of healing in bone fractures by placing muscle tissue over the surrounding lesion.^73^ Since these findings, along with other work in this area,^33^ muscle and bone have been established as endocrine organs secreting multiple growth factors and anabolic and catabolic molecules, which may influence both tissues.^19^ Collectively, the role of myokines, osteokines, and adipokines in bone and muscle cross talk is of increasing interest in the pathogenesis of osteosarcopenia (Figure 2).^6^

The musculoskeletal system interacts via autocrine, paracrine, and endocrine signaling, and multiple communication pathways in the muscle‐bone unit have been identified. Bone receives anabolic signals from muscle and vice versa. A number of myokines, upregulated by muscle contraction, play roles in bone formation and resorption.^72^ For instance, myostatin, a member of the transforming growth factor superfamily, inhibits muscle growth via inhibition of myoblast proliferation.^74^ In both animal and human models, myostatin absence or deficiency increases muscle mass and function,^75^, ^76^ whereas administering myostatin induces muscle atrophy via a downregulation of gene expression.^77^ Myostatin gene deficiency also increases osteogenic differentiation of stromal cells, enhancing bone repair and density.^78^ In addition, systemic administration of a myostatin decoy receptor (ACVR2B‐Fc) increases muscle and bone mass.^48^

More growth factors secreted by myotubes, notably IGF‐1 and fibroblast growth factor‐2 (FGF‐2), are anabolic to not only muscle, but also bone.^48^, ^79^ In vivo and in vitro models indicate IGF‐1 and FGF‐2, localized in the periosteum, stimulate osteoblastogenesis and bone remodelling.^80^ Moreover, interleukin (IL)‐6, another myokine, upregulates bone resorption during chronic low‐grade inflammation released by immune cells and hepatocytes.^81^ Several other myokines, including osteoglycin, irisin, osteonectin, and IL‐15, play roles in muscle‐bone cross talk.^82^

In the opposite direction, bone secretes growth factors known to influence muscle. Of those, osteocalcin, a key player in bone energetics, upregulates pancreatic β‐cells and insulin secretion in muscle, correlating with leg strength in older women.^83^ However, a recent cross‐sectional study^79^ of middle‐aged and older adults found that serum IGF‐1, but not sclerostin or osteocalcin, was associated with muscle mass and function as well as bone metabolism in this cohort. Stromal cells in bone marrow also stimulate myoblast proliferation via the paracrine release of vascular endothelial growth factor,^84^ providing further evidence of endocrine cross talk between these organs.

Recent advancements in the field have deciphered the role of fatty infiltration in metabolically active tissues.^85^ We^6^, ^35^, ^36^, ^86^ and others^37^ have documented the detrimental effects of intramuscular and bone marrow fat, which are toxic to myocytes, osteoblasts and osteocytes in the vicinity, driven by the secretion of inflammatory cytokines. In humans, elevated circulating concentrations of pro‐inflammatory adipocytokines, notably IL‐6, tumor necrosis factor alpha, adiponectin, and leptin, have been found in sarcopenic and osteopenic populations,^87^, ^88^ and are associated with systemic and local lipotoxicity.^89^

Among the fatty acids released from adipocytes, palmitic acid (PA) is the most prevalent in marrow adipocytes in vitro and in vivo.^90^, ^91^ We previously reported the lipotoxic effects of PA, which compromised osteoblast,^92^, ^93^and osteocyte,^94^ function, and survival. Interestingly, a similar lipotoxic effect of PA has been observed in myotubes,^95^ which led us to investigate the effects of PA inhibitors. Indeed, we treated ovariectomized mice with cerulenin, an inhibitor of fatty acid synthase, and found it rescued osteoblasts from apoptosis while recovering their bone‐forming potential.^96^ In another study, we incubated human osteoblasts with rapamycin—a macrolide compound that regulates the mammalian target of rapamycin (mTOR) signaling pathway and has been proposed as an important regulator of aging in the geroscience field^97^in the presence of lipotoxic PA, and found that rapamycin attenuated PA‐induced apoptosis,^98^ thus suggesting that rapamycin administration could potentially be utilized as a bone‐protecting strategy in osteoporotic bone. Whether the same effect is observed in muscle is a subject of ongoing research.

## 3 TRANSLATIONAL GEROSCIENCE AND OSTEOSARCOPENIA

Recently, the American Geriatrics Society highlighted the importance of translational geroscience as an integrated approach to several age‐related diseases.^99^ The consensus concluded that “the study of geroscience therapeutics is mostly in early‐stage, first‐in‐human, or proof‐of‐concept clinical trials.”

Although a similar case is occurring regarding pharmacological approaches to osteosarcopenia, where a combined pharmacological approach is still missing,^15^, ^100^ there are current non‐pharmacological approaches and potential therapeutic targets that offer a promissory future to the field.

### 3.1 Non‐pharmaceutical treatments

Resistive‐based exercise remains the most promising strategy to combat osteosarcopenia. In sarcopenic populations, resistance exercise (RE) increases muscle mass, strength, and function,^101^, ^102^, ^103^ while Cochrane reviews highlight small but clinically relevant increases in bone density following impact exercise in osteoporotic patients.^104^ The efficacy of RE in preventing falls in older persons is also established^105^ and in obese adolescents, exercise prescription reduced ectopic fat in muscle and bone marrow,^106^ although these effects need clarifying in osteosarcopenic elders. Preserving muscular strength is protective against all‐cause mortality too, including incidence of cancer^107^ and heart disease.^108^ At present, the International Clinical Practice Guidelines for Sarcopenia recommend RE as a primary treatment option^109^ and consensus groups promote impact exercise as a safe and effective way to avert bone loss.^104^

In relation to nutritional status, a higher intake of protein (including leucine and its end products, such as beta‐hydroxy‐beta‐methylbutyrate) is needed to offset declining protein synthesis rates in sarcopenic muscle.^31^ Indeed, chronic protein supplementation augments the beneficial effects of RE^18^ and the PROT‐AGE study group advocates at least 1.2‐1.5 g/kg/d of protein (with 2.5‐3 g of leucine per meal) for sarcopenic individuals^110^ with no adverse effects reported. Cross‐sectional data support a higher intake of protein for the preservation of bone mass as well,^54^ particularly under conditions of adequate calcium intake.^111^ However, recent prospective studies show conflicting findings,^112^, ^113^ highlighting the need for further randomized clinical trials.

With aging, vitamin D bioavailability declines. This, explained by a combination of lack of sunlight exposure, cultural factors, dietary habits, and alterations in the expression of the vitamin D receptor, increases the risk of osteosarcopenia. Current guidelines suggest 800 IU (20 μg) of vitamin D from all dietary sources is required to offset falls in older adults.^49^ Higher doses of calcium associate with greater bone mineral density too,^114^ and given bone is the largest depot of calcium in the body, it seems imperative to maintain sufficient intake with age, despite the debate of this micronutrient in lowering fracture risk.^115^ Creatine is another nutrient that has consistently been shown to enhance muscle mass, strength, and function^116^, ^117^ and may be a promising strategy to preserve bone microarchitecture and strength,^118^ although investigations have largely been conducted in healthy older adults.

Given these findings, there is sufficient evidence to recommend adequate protein, vitamin D, calcium, and creatine intake as the first line of treatment for osteosarcopenia. However, a clear drawback of current research is the limited number of trials examining the effect of dual therapies in osteosarcopenic patients. Moreover, the synergistic effects of exercise and nutrition on circulating hormones, growth factors, and inflammatory cytokines, which are implicated in the pathophysiology of osteosarcopenia, require further investigation.

## 4 PHARMACOLOGICAL APPROACH: TARGETING THE PILLARS OF AGING IN OSTEOSARCOPENIA

With the increasing understanding of the mechanisms of osteosarcopenia, new therapeutic approaches or repurposing of current compounds is translating into phase II and III trials (as summarized in Fatima et al^119^ and Zanker and Duque^120^). 

### 4.1 Denosumab

Denosumab is a humanized monoclonal antibody to receptor activator of nuclear factor‐κB ligand (RANKL). The binding of RANKL to the RANK receptor on osteoclast is responsible for its activation, differentiation, and osteoclastic action. By blocking RANKL, denosumab blocks the activation of osteoclasts, thus protecting bone from resorption and increasing bone mass.

Interestingly, results from the FREEDOM trial,^121^ which demonstrated an effect of denosumab on fracture prevention, also indicated that the denosumab‐treated group experienced fewer falls (4.5%) compared to the untreated group (5.7%; *P *= .02). A recent study by Bonnet et al^122^ tested the effect of denosumab on animals and humans (postmenopausal women). The authors report that RANKL deteriorates, while its inhibitor improves, muscle strength and insulin sensitivity in osteoporotic mice and humans, and conclude that in addition to its role as a treatment for osteoporosis, denosumab could represent a novel therapeutic approach for sarcopenia and thus for osteosarcopenia. Further studies looking at the direct effect of denosumab on muscle mass and function are still needed.

### 4.2 Testosterone and selective androgen receptor modulators

As previously mentioned, testosterone levels decrease with age and are considered an important cause of osteosarcopenia. Although results from clinical trials testing the effect of testosterone administration on bone and muscle mass and on reduction of adverse events (ie, falls and fractures) have been mostly disappointing, selective androgen receptor modulators (SARMs), which could have an effect on sarcopenia,^123^ could also have a synchronic effect on osteoporotic bone. In a recent phase II trial, VK5211, an oral non‐steroid SARM, showed a significant increase in lean muscle mass and non‐significant improvement in 6‐minute walk test in the treatment group at 12 weeks (R. Ristic, V. Harhaji and V. Sirbu, unpublished data, September 2018). Additionally, the treatment group showed a significant improvement in procollagen type 1 N propeptide (P1NP), a marker of bone formation, suggesting a dual effect on bone and muscle; this is an exciting possibility for the potential treatment of osteosarcopenia. In addition, two major trials—“The Testosterone Trial in Older Men” (http://www.clinicaltrials.gov) and “T4DM” (http://www.t4dm.org.au)—are underway and aim to clarify the role of testosterone in the management of osteosarcopenia.

### 4.3 Anti‐myostatin antibodies

Although anti‐myostatin antibodies have been proposed as the “Holy Grail” for muscle, bone, and fat, the results of clinical trials have not been encouraging.^124^ In aged mice, anti‐myostatin antibodies increased muscle mass and strength.^125^ A phase II trial in older adults (aged ≥75 years) with a history of fall showed that anti‐myostatin antibodies increased the lean body mass and mildly improved functional measures associated with muscle strength.^126^ Regarding the role of myostatin antibodies in improving bone health, data from animal studies indicate that in combination with resistant exercise, myostatin antibodies improved bone mass;^127^ however, whether the same effect is also observed in humans still requires future clinical trials.

### 4.4 Rapamycin and regulation of mTOR

As mentioned above, IGF‐1 and IL‐6 play important roles in the muscle‐bone cross talk. These are important regulators of the Akt/mTOR pathway, which regulates mRNA translation and protein synthesis in skeletal muscle while also regulating bone remodeling.^128^ Therefore, pharmacological modulation of the Akt/mTOR pathway could become a novel approach to osteosarcopenia. Considering that rapamycin is a natural product that inhibits mTOR with high specificity while also regulating other age‐affected processes, such as autophagy and apoptosis, which are also highly prevalent in osteoporosis and sarcopenia, it could become an attractive therapeutic approach to osteosarcopenia. By demonstrating that rapamycin affects palmitate‐induced lipotoxicity in osteoblasts by modulating apoptosis and autophagy in vitro,^98^ we then proposed that rapamycin‐associated therapies could, potentially, be targeted for specific roles in osteoporosis and sarcopenia. Future animal studies will be required before progressing into clinical trials.

### 4.5 Go for the fat: Targeting adipocyte products to treat osteosarcopenia

Fat infiltration of muscle and bone is one of the hallmarks in osteosarcopenia. Increasing the number of local adipocytes within the bone marrow and within and between muscle fibers (a phenomenon that is independent of body mass index^36^) results in secretion of lipotoxic factors (mostly PA), which has a toxic effect on cells in their vicinity.^36^ Based on this evidence, we hypothesized that inhibition of fatty acid synthase would have a beneficial effect on bone and muscle in vivo. Osteoporotic mice treated with cerulenin showed a recovery in their bone mass associated with higher levels of bone formation,^96^ probably due to a less lipotoxic environment within the bone marrow. Similar findings have been obtained in muscle cells treated with palmitate and cerulenin in vitro and in sarcopenic mice in vivo (A. Al Saedi and G. Duque, unpublished data, August 2019). However, further experiments specifically targeting fatty acid synthase in muscle and bone adipocytes are still required.

## 5 SUMMARY

Osteosarcopenia is a prevalent musculoskeletal syndrome conferring an increased risk of falls, fractures, and hospitalizations. Findings from basic and clinical sciences suggest that osteosarcopenia is an optimal target for translational geroscience research since multiple pathways illustrating cross talk among these tissues and involving the pillars of aging have been identified. At present, resistance exercise, as well as dietary protein, vitamin D, calcium, and creatine intake are the only evidence‐based strategies to mitigate osteosarcopenia. However, individuals suffering from chronic diseases are often resistant to exercise and dietary interventions. In these cases, pharmaceutical treatments with dual effects on muscle and bone are required. Current (ie, denosumab, SARMs) and future (ie, anti‐myostatin antibodies, rapamycin, fatty acid synthase inhibitors) compounds have shown promissory dual effects on muscle and bone that deserve further exploration. Finally, development of robust biomarkers for osteosarcopenia with the capacity to diagnose this syndrome, predict adverse outcomes, and monitor treatment response are highly needed.

## CONFLICTS OF INTEREST

There are no conflicts of interest to be reported by the authors of this study.
